# LncRNA *OIP5-AS1/cyrano* suppresses GAK expression to control mitosis

**DOI:** 10.18632/oncotarget.17219

**Published:** 2017-04-19

**Authors:** Jiyoung Kim, Ji Heon Noh, Seung-Kyu Lee, Rachel Munk, Alexei Sharov, Elin Lehrmann, Yongqing Zhang, Weidong Wang, Kotb Abdelmohsen, Myriam Gorospe

**Affiliations:** ^1^ Laboratory of Genetics and Genomics, National Institute on Aging-Intramural Research Program, NIH, Baltimore, MD 21224, USA

**Keywords:** lncRNA, proliferation, mitosis

## Abstract

Some long noncoding RNAs (lncRNAs) can regulate gene expression programs, in turn affecting specific cellular processes. We sought to identify the mechanism through which the lncRNA *OIP5-AS1*, which is abundant in the cytoplasm, suppressed cell proliferation. Silencing of *OIP5-AS1* in human cervical carcinoma HeLa cells triggered the appearance of many aberrant (monopolar, multipolar, misaligned) mitotic spindles. Through a combination of approaches to pull down mRNAs bound to *OIP5-AS1* and identify proteins differentially expressed when *OIP5-AS1* was silenced, we identified a subset of human cell cycle regulatory proteins encoded by mRNAs that interacted with *OIP5-AS1* in HeLa cells. Further analysis revealed that *GAK* mRNA, which encodes a cyclin G-associated kinase important for mitotic progression, associated prominently with *OIP5-AS1*. The interaction between these two transcripts led to a reduction in *GAK* mRNA stability and GAK protein abundance, as determined in cells in which *OIP5-AS1* levels were increased or decreased. Importantly, the aberrant mitotic cell division seen after silencing *OIP5-AS1* was partly rescued if GAK was simultaneously silenced. These findings indicate that the abnormal mitoses seen after silencing *OIP5-AS1* were caused by an untimely rise in GAK levels and suggest that *OIP5-AS1* suppresses cell proliferation at least in part by reducing GAK levels.

## INTRODUCTION

To maintain cell homeostasis, gene expression patterns are strongly regulated post-transcriptionally, through mechanisms like pre-RNA splicing and maturation, and mRNA transport, stability, storage, editing and translation [1–4]. Besides RNA-binding proteins (RBPs), the major post-transcriptional regulatory factors are noncoding RNAs (ncRNAs), a large class that includes microRNAs and long noncoding RNAs (lncRNAs). Through their impact on protein expression programs, ncRNAs have been implicated in cellular processes that broadly influence physiological adaptation and diseases like cancer, cardiovascular function, and neurodegeneration [5–10].

The development of high-throughput RNA sequencing has revealed that thousands of ncRNAs are transcribed from >90% of the human genome and are expressed selectively in some cell types, as well as in response to specific developmental and environmental conditions [7–16]. LncRNAs are defined as noncoding transcripts longer than 200 nucleotides that generally do not encode proteins, although some of them are indeed translated [12, 15, 17]. They can reside in the nucleus and the cytosol and vary widely in their length, genomic locus of origin, interacting partners, and mechanisms of action. Some lncRNAs critically alter gene transcription by influencing chromosome organization, epigenetic changes, and transcription factor recruitment. Other lncRNAs affect gene expression post-transcriptionally by altering mRNA splicing, translation, and stability, as well as the availability of RBPs and other mRNA-binding factors [5].

Numerous lncRNAs have been implicated in carcinogenesis. As reviewed recently by Evans and colleagues [18], a vast number of lncRNAs have been associated with a wide range of human malignancies, including breast, ovarian, prostate, lung, thyroid, and kidney cancers. Through their impact on protein expression programs at the transcriptional, post-transcriptional, and post-translational levels, lncRNAs were found to affect cancer cell homeostasis. Indeed, several thousand lncRNAs have been reported to control one or several major cancer traits, such as cell proliferation, the hypoxia response, the TP53 pathway, cell senescence, telomere maintenance, signaling through hormone receptors, and the apoptotic response [18–21].

We recently investigated the human lncRNA *OIP5-AS1*, which is transcribed in the antisense (AS) direction from the same gene that encodes Opa-interacting protein 5 (*OIP5*), a pro-tumorigenic protein overexpressed in many cancers. *OIP5-AS1* was initially identified as *cyrano* in zebrafish [22], where it was found to play a key role in early development of the central nervous system. In HeLa cells, *OIP5-AS1* was found to suppress cell proliferation at least in part by associating with the RBP HuR and reducing its availability to target mRNAs including those that encode cyclins A and D1 (CCNA2 and CCND1) and SIRT1. Accordingly, these proteins were more highly expressed when *OIP5-AS1* levels declined and were suppressed when *OIP5-AS1* was overexpressed [23].

To investigate more comprehensively *OIP5-AS1* actions, particularly its anti-proliferative activity, we focused on the observation that silencing *OIP5-AS1* led to the appearance of aberrant (monopolar, multipolar, misaligned) spindles. We performed affinity pulldown analysis to identify *OIP5-AS1*-associated mRNAs and *OIP5-AS1* silencing to uncover cell cycle proteins differentially regulated by *OIP5-AS1*. These analyses revealed that *OIP5-AS1* associated with *GAK* mRNA, which encodes GAK, a 144-kDa Ser/Thr kinase associated with cyclin G and required for metaphase transition [24, 25]. In turn, *OIP5-AS1* lowered *GAK* mRNA stability and reduced GAK protein abundance. Our results suggested that *OIP5-AS1* contributed to the proper chromosome segregation during mitosis by maintaining low GAK levels. This hypothesis was supported by the discovery that the aberrant mitoses seen after *OIP5-AS1* were partly rescued by concomitant GAK silencing, indicating that GAK was an effector of the influence of *OIP5-AS1* on cell proliferation.

## RESULTS

### LncRNA *OIP5-AS1/cyrano* is required for proper mitotic cell division

**Figure 1:**
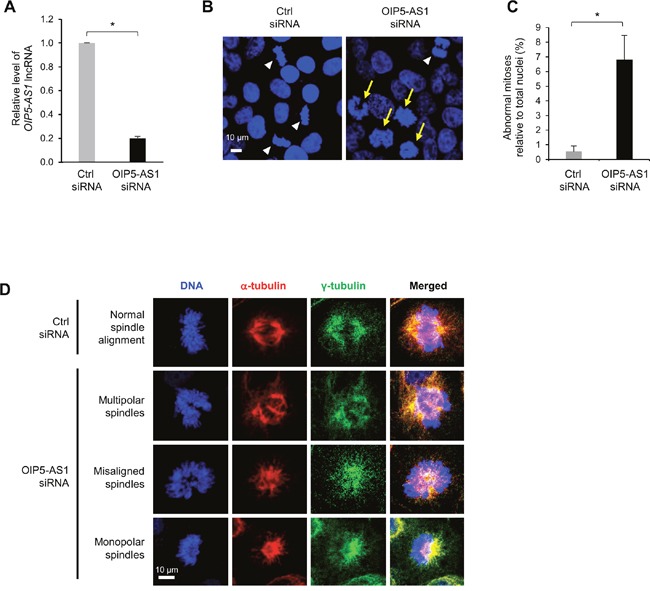
Silencing *OIP5-AS1* induces abnormal mitotic cell division **(A)** HeLa cells were transfected with a control (Ctrl) siRNA or siRNA directed to *OIP5-AS1*; 72 h later, total RNA was isolated and the levels of *OIP5-AS1* were measured by RT-qPCR analysis. Data were normalized to levels of *18S* rRNA, also measured by RT-qPCR analysis. Data represent the means of three independent experiments +S.E.M.; *, *p*<0.05. **(B)** In HeLa cells that were processed as described in (A), DNA was visualized with Hoechst 33342. Yellow arrows, abnormal mitotic chromosomes; white arrowheads, normal mitotic chromosomes. **(C)** Abnormal mitoses relative to total nuclei (%) were quantified from 4 different experiments; data represent the means of four independent experiments + S.E.M. **(D)** In HeLa cells that were processed as described in (A), DNA was visualized using Hoechst 33342, spindle microtubules using anti-α-tubulin, and centrosomes using anti-γ-tubulin. Examples of multipolar, misaligned, and monopolar spindles in the OIP5-AS1 siRNA populations are shown. In (B, D), data are representative of three independent experiments; images were collected using Zeiss LSM 710 microscope.

We previously reported that human lncRNA *OIP5-AS1* suppressed cell proliferation [23]. We investigated this phenotype further by silencing *OIP5-AS1* in human cervical carcinoma HeLa cells using small interfering (si)RNA directed at *OIP5-AS1* or control (Ctrl) siRNA (Figure 1A). By 72 h after siRNA transfection, HeLa cells exhibited aberrant chromosome condensation during mitosis, as determined by staining DNA using Hoechst 33342 and visualization using fluorescence microscopy (Figure 1B; white arrowheads, normal mitoses; yellow arrows, abnormal mitoses). Abnormal mitotic cells were counted in multiple fields and calculated relative to total nuclei; in total, 322 and 485 nuclei were assessed in Ctrl siRNA and *OIP5-AS1* siRNA groups, respectively (Figure 1C). The abnormal mitotic spindle morphologies were further analyzed by staining with Hoechst 33342 as well as with antibodies that recognized α-tubulin (TUBA1) to visualize microtubules, and γ-tubulin (TUBG1) to visualize the centrosomes. As shown, mitotic cells in the control group showed normal alignment of chromosomes at the metaphase plate (Figure 1D, Ctrl siRNA). By contrast, in *OIP5-AS1*-silenced mitotic cells, chromosomes were disorganized and less condensed, with misaligned mitotic spindles and abnormally polarized microtubules (Figure 1D, OIP5-AS1 siRNA). Silencing *OIP5-AS1* caused three distinguishable abnormalities in the arrangement of mitotic chromosomes; among them, multipolar spindles were most common, but misaligned/unelongated spindles and monopolar spindles were also observed (Figure 1D, OIP5-AS1 siRNA). These observations support the notion that *OIP5-AS1* plays a critical role in the alignment of chromosomes and microtubules during metaphase and suggest that *OIP5-AS1* is required for proper mitotic spindle formation.

### Identification of cell cycle proteins affected by *OIP5-AS1*, encoded by *OIP5-AS1*-interacting mRNAs

**Figure 2:**
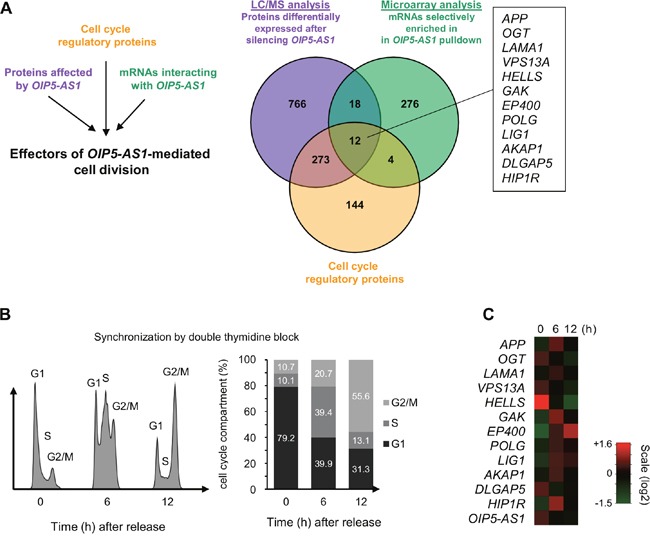
Cell cycle proteins regulated by *OIP5-AS1*, encoded by *OIP5-AS1*-interacting mRNAs **(A)** Schematic of the three-pronged approach used to identify systematically the mediators of cell division by *OIP5-AS1*. *Purple*, in cells that were processed as described in Figure 1A, LC/MS analysis was carried out to identify proteins differentially expressed (higher or lower) in *OIP5-AS1*-silenced cells (Supplementary Table 1). *Green*, in HeLa cells transfected with two vectors, one that expressed *MS2-OIP5-AS1* chimeric RNA and one that expressed the fusion protein MS2-GST, pulldown analysis was carried out and mRNAs interacting with *MS2-OIP5-AS1* were identified by microarray analysis (GSE93551). *Orange*, from among a list of cell cycle regulatory proteins, we identified 12 mRNAs (box) encoding proteins implicated in controlling cell proliferation, enriched in *MS2-OIP5-AS1* pulldown and showing changed expression upon *OIP5-AS1* silencing. **(B)** U2OS cells were synchronized by double thymidine block; 6 and 12 h following release, cells were collected for FACS and microarray analyses. The cell cycle distribution profiles (*left*) and the percentage of cells in the G1, S, and G2/M compartments (*right*) following release are indicated. **(C)** Microarray analysis in cells processed as described in (B) to identify differentially expressed transcripts. Heat-map analysis focused on the 12 mRNAs identified in panel (A) plus *OIP5-AS1*. Scale intensity is indicated.

To identify systematically the possible mediator(s) of the mitotic phenotype observed after silencing *OIP5-AS1*, we devised a three-pronged strategy. First, we sought to identify *OIP5-AS1*-regulated proteins by silencing *OIP5-AS1* and performing LC/MS proteomic analyses to find proteins abundant in each cell population (Figure 2A, purple). This analysis revealed about 1000 proteins differentially expressed (twofold higher or twofold lower) in cells in which *OIP5-AS1* was silenced (Supplementary Table 1). Second, we identified mRNAs associated with *OIP5-AS1* by transfecting cells with two expression vectors, one that transcribed the chimeric RNA *MS2-OIP5- AS1* and one that expressed a MS2-GST fusion protein. GSH beads were then used to pull down the MS2 complex from cell lysates, thereby capturing *MS2*-*OIP5-AS* and molecules bound to it; the associated mRNAs were then identified by microarray analysis as described (GSE93551) (Figure 2A, green). Finally, from the intersection of proteins showing differential abundance after *OIP5-AS1* silencing (purple), and whose cognate mRNAs associated with *OIP5-AS1* (green), we searched for known proteins implicated in regulating cell division (Figure 2A, orange) and discovered 12 mRNAs which met all three criteria (box).

We sought to gain further insight into the function of *OIP5-AS1*-interacting mRNAs given the phenotype observed in *OIP5-AS1*-silenced cells. To this end, we synchronized human osteosarcoma U2OS cells using double thymidine block to stop cell cycle progression at the end of the G1 phase. This cell line was chosen because it can be synchronized robustly and provides a more reliable model of cell cycle progression than HeLa cells. FACS analysis indicated that 6 h after release from synchronization most cells were transitioning through the S phase, and 12 h later they were highly abundant in the G2/M compartments (Figure 2B). At these time points, RNA was collected and studied using microarrays. Interestingly, some mRNAs showed expression patterns similar to that seen for *OIP5-AS1* (e.g., *OGT*, *HELLS*, and *DLGAP5* mRNAs), while other mRNAs showed expression patterns opposite to that of *OIP5-AS1* (e.g., *APP*, *GAK*, *EP400*, and *HIP1R* mRNAs) (Figure 2C). To test if the interaction of mRNAs and *OIP5-AS1* is conserved between U2OS and HeLa cells, we repeated the MS2 pulldown assay after transfecting HeLa and U2OS cells with the plasmids described above [23]. As shown in Supplementary Figure 1, some mRNAs (e.g., *GAK*, *HELLS*, *POLG* and *SIRT1* mRNAs) showed enrichment in *OIP5-AS1* pulldown in both cell types, while other mRNAs did not (e.g., *VPS13A*, *APP* and *EP400*, not shown). These findings suggested that *OIP5-AS1* might influence the expression levels of some bound target mRNAs and decrease the abundance of other bound mRNAs, and this influence appears at least partially conserved in two human cancer cells lines.

### *OIP5-AS1* interacts with *GAK* mRNA, lowering *GAK* mRNA stability and GAK expression levels

**Figure 3:**
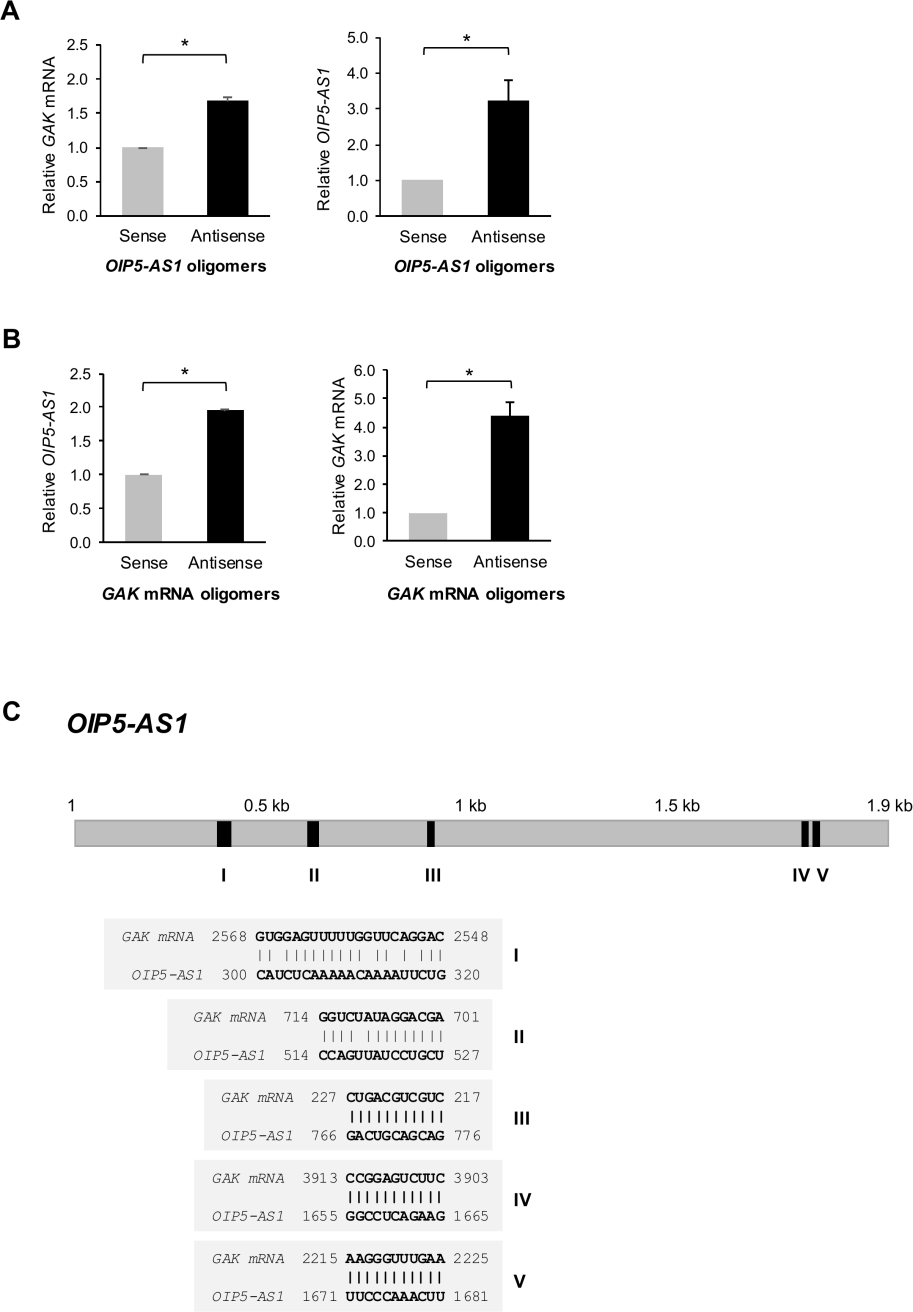
*OIP5-AS1* interacts with *GAK* mRNA **(A)** HeLa cell lysates were incubated with 3′ end-biotin-labeled oligomers complementary to *OIP5-AS1* (*antisense*) or reverse complementary control oligomers (*sense*). Following pulldown using streptavidin beads and RNA isolation, the levels of *GAK* mRNA (*left*) and *OIP5-AS1* (*right*) were assessed by RT-qPCR analysis and normalized to the levels of *GAPDH* mRNA. **(B)** HeLa cell lysates studied as in (A) using 3′ end-biotin-labeled oligomers complementary to *GAK* mRNA (*antisense*) or reverse complementary control oligomers (*sense*). The levels of *OIP5-AS1* (left) and *GAK* mRNA (right) were assessed by RT-qPCR analysis and normalized to the levels of *GAPDH* mRNA. In (A, B), data represent the means + S.E.M. from three independent experiments; *, p<0.05. **(C)** Regions of complementarity (shaded segments I-V) between *OIP5-AS1* and *GAK* mRNA.

We identified *GAK* mRNA as the most prominent *OIP5-AS1*-interacting mRNAs that encoded a protein (GAK) that was both suppressed by *OIP5-AS1* and implicated in mitotic progression. Accordingly, we set out to look in detail at this interaction and investigate its functional consequences. We designed 3′-end biotinylated antisense DNA oligomers complementary to *OIP5-AS1* as well as biotinylated control sense oligomers, incubated them with whole-cell lysates, and pulled them down, along with interacting RNAs using streptavidin magnetic beads. As measured by reverse transcription (RT) followed by real-time quantitative (q)PCR analysis, *GAK* mRNA was enriched 1.6-fold (Figure 3A, *left*) in the samples that selectively pulled down *OIP5-AS1* compared with the sense control oligomers (Figure 3A, *right*). Similarly, we designed 3′-end biotinylated antisense oligomers complementary to *GAK* mRNA as well as biotinylated control sense oligomers, incubated them with whole-cell lysates, and used streptavidin magnetic beads to pull them down along with any interacting RNAs. RT-qPCR analysis of the RNA in the pulldown material revealed that *OIP5-AS1* was enriched twofold in the samples that selectively pulled down *GAK* mRNA, relative to what was pulled down using sense control oligomers (Figure 3B). Several regions of complementarity between *OIP5-AS1* and *GAK* mRNA were identified (Figure 3C). Neither the individual sites of complementarity nor combinations of sites tested appeared fully responsible for recapitulating the specific RNA interactions (not shown), suggesting that perhaps larger complexes, possibly involving other RBPs, might be necessary for efficient interaction between the two RNAs.

**Figure 4:**
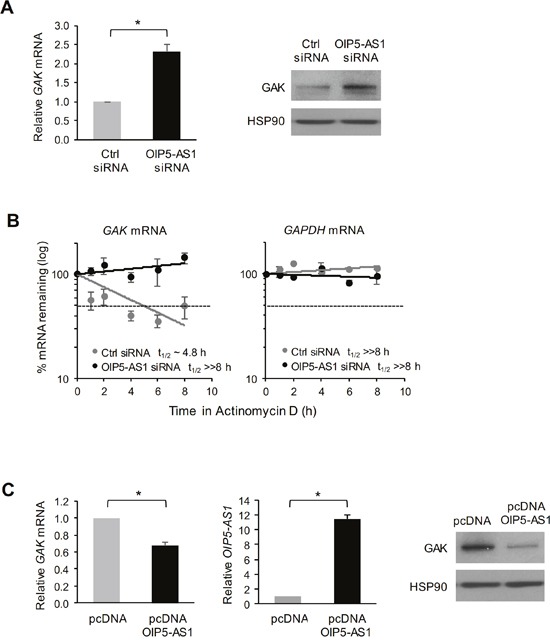
Impact of *OIP5-AS1* on *GAK* mRNA stability and GAK protein expression levels **(A)** Seventy-two hours after transfecting HeLa cells with either Ctrl siRNA or OIP5-AS1 siRNA, the levels of *GAK* mRNA were measured by RT-qPCR analysis (*left*) and the levels of GAK protein and loading control HSP90 were assessed by Western blot analysis (*right*). **(B)** In cells processed as described in (A), the half-life of *GAK* mRNA was measured by adding actinomycin D and calculating the time needed for *GAK* mRNA to reach one half (t_1/2_) of its abundance at time 0; the half-life of a stable transcript, *GAPDH* mRNA, which encodes a housekeeping protein, was measured in control experiments. RNA levels at each time point were normalized to *18S* rRNA levels. **(C)** Forty-eight hours after transfection of pcDNA-OIP5-AS1 to overexpress *OIP5-AS1*, the levels of *GAK* mRNA (left graph) and *OIP5-AS1* (right graph) were measured by RT-qPCR analysis (*left*) and the levels or GAK protein and loading control HSP90 were assessed by Western blot analysis (*right*). In (A-C), data represent the means and S.E.M. from three independent experiments. In (A, C), *, *p*<0.05.

To investigate the consequences of the interactions between *GAK* mRNA and *OIP5-AS1*, we silenced *OIP5-AS1* in HeLa cells and harvested the cells 72 h later to determine the levels of *GAK* mRNA and GAK protein using RT-qPCR and Western blot analyses, respectively. As shown in Figure 4A, silencing *OIP5-AS1* increased the levels of *GAK* mRNA and GAK protein significantly. The increase in *GAK* mRNA was due at least in part to stabilization of *GAK* mRNA, as determined by measuring *GAK* mRNA stability following treatment with actinomycin D to stop *de novo* RNA transcription in cells and measuring the time required for *GAK* mRNA to reach 50% of its initial abundance before adding actinomycin D. As shown in Figure 4B, *GAK* mRNA had a substantially longer half-life (>> 8 h) in cells with silenced *OIP5-AS1* than in control cells (in which the half-life was ∼4.8 h). The half-life of *GAPDH* mRNA, a stable control transcript encoding a housekeeping protein, was not affected by silencing *OIP5-AS1* (Figure 4B). Conversely, overexpression of *OIP5-AS1* by transfection of the expression vector pcDNA-OIP5-AS1 lowered *GAK* mRNA levels and GAK abundance, as determined by using RT-qPCR and Western blot analyses, respectively (Figure 4C). Together, these findings indicate that *OIP5-AS1* binds *GAK* mRNA and negatively regulates *GAK* mRNA stability, in turn lowering the steady-state levels of *GAK* mRNA and GAK protein.

### Abnormal mitotic cells in silenced *OIP5-AS1* were restored by *GAK* silencing

**Figure 5:**
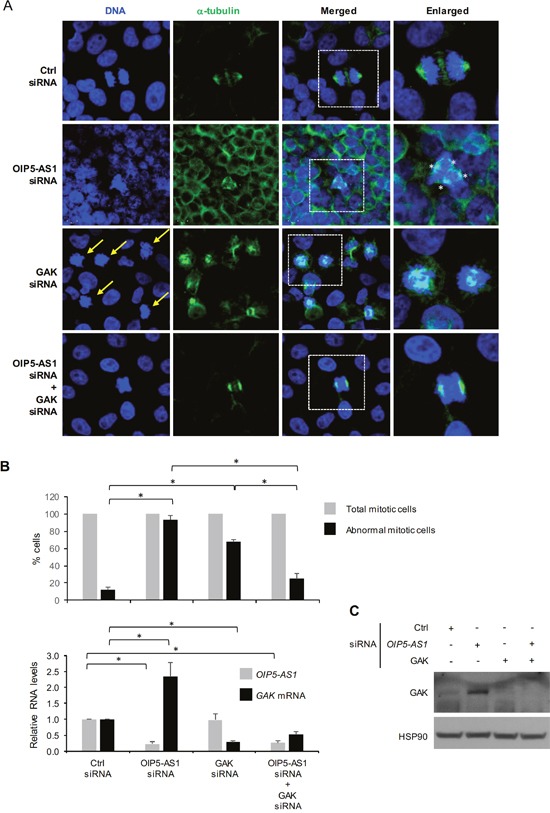
Impact of silencing GAK on abnormal mitoses in *OIP5-AS1* silenced cells **(A)** Seventy-two hours after transfecting HeLa cells with Ctrl siRNA, OIP5-AS1 siRNA, GAK siRNA, or with both OIP5-AS1 + GAK siRNAs, DNA was visualized using Hoechst 33342 and spindle microtubules using anti-α-tubulin. Yellow arrows, abnormal mitotic chromosomes. **(B, C)** In cells that were processed as described in (A), 18-42 mitotic nuclei (in 3-6 fields chosen randomly in 3 independent experiments) were counted and the number of normal and abnormal mitoses calculated (B, *top*), RNA was extracted and the levels of *OIP5-AS1* and *GAK* mRNA was quantified by RT-qPCR analysis (B, *bottom*), or protein was extracted (C) and the levels of GAK and loading control HSP90 were assessed by Western blot analysis. In (B), data represent the means and S.E.M. from three independent experiments; *, *p*<0.05. The data in (A, C) are representative of three independent experiments.

**Figure 6:**
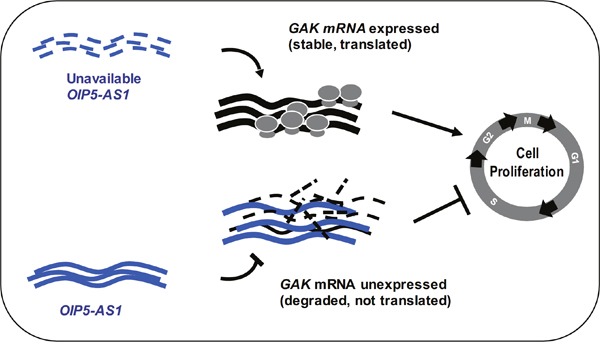
Schematic Model proposed to explain our findings. Low *OIP5-AS1* levels causes a rise in GAK expression levels and accelerates cell division, while elevated *OIP5-AS1* suppresses GAK abundance and inhibits cell division. The increase in GAK abundance when *OIP5-AS1* levels decline is due, at least in part, to the stabilization of the *GAK* mRNA when *OIP5-AS1* is silenced.

Given that silencing *OIP5-AS1* triggered a rise in GAK levels and dysfunctional mitoses, we investigated if the mitotic impairment elicited by the reduction in *OIP5-AS1* was mediated at least in part by the higher GAK abundance. GAK had been implicated in the assembly of the mitotic spindle [25], and therefore we hypothesized that the higher GAK levels resulting from lowering *OIP5-AS1* might contribute to causing abnormal mitoses. To test this hypothesis, we performed a rescue experiment in which the impact of *OIP5-AS1* silencing on the defective mitotic phenotype was compared between cells expressing high GAK levels and cells in which GAK expression was ectopically silenced. As shown in Figure 5A and quantified in Figure 5B, the aberrant mitoses (particularly multipolar spindles) seen after silencing *OIP5-AS1* were largely rescued after simultaneously silencing *GAK* mRNA (OIP5-AS1 siRNA + GAK siRNA group), although simply silencing *GAK* mRNA also increased the appearance of some abnormal mitoses, as reported [25]. Silencing *OIP5-AS1* elevated *GAK* mRNA and GAK protein levels (Figure 5B and 5C), although GAK siRNA potently silenced GAK expression regardless of *OIP5-AS1* abundance. Taken together, our results indicate that *OIP5-AS1* helps to elicit proper mitotic progression at least in part by binding *GAK* mRNA and ensuring low production of GAK protein (Figure 6).

## DISCUSSION

In this study, we sought to identify systematically the mediators whereby *OIP5-AS1* represses cell division. We previously found that *OIP5-AS1* sequestered the RBP HuR, which reduced the availability of HuR to target mRNAs encoding cyclins A and D1 (CCNA2 and CCND1) and consequently lowered their expression levels, resulting in reduced proliferation [23]. To elucidate comprehensively the mediators of *OIP5-AS1*-regulated cell division, we used a strategy that identified *OIP5-AS1*-interacting mRNAs. We then selected those mRNAs encoding cell cycle regulatory proteins that showed altered levels after modulating *OIP5-AS1* abundance. From the short list of 12 mRNAs that met these criteria, we focused our further analysis on *GAK* mRNA, as reduced GAK levels had been linked to appearance of abnormal mitoses [25] and elevated GAK levels were reported in several different cancers [26, 27].

Molecular analysis of the mechanisms through which *OIP5-AS1* regulated GAK expression revealed that *OIP5-AS1* and *GAK* mRNA shared stretches of complementarity that might contribute to the association of these two RNAs, although it is likely that other factors, such as RBPs, participate in this interaction. The observed association of the two RNAs (Figure 3) was likely to occur in the cytoplasm, in keeping with the finding that *OIP5-AS1* was present in this compartment in HeLa cells [23]. We propose that the partial complementarity between *OIP5-AS1* and *GAK* mRNA led to degradation of *GAK* mRNA, and conversely that silencing *OIP5-AS1* increased *GAK* mRNA half-life. Earlier examples of decay-promoting lncRNAs include the ½sbs-RNAs, which accelerate the degradation of mRNAs with which they shared partial complementarity [28]. Whether *OIP5-AS1* influenced additional levels of regulation of GAK expression, such as the cytoplasmic export, editing, storage, or translation of *GAK* mRNA, remains to be investigated.

Although *OIP5-AS1* deficiency had been linked to several diverse cellular processes, it was apparent that GAK, a ubiquitous protein, might be implicated in events involving the DNA damage response, cell cycle checkpoints, and cell survival. However, the increase in GAK levels may also be responsible for other distinct phenotypes attributed to the reduction in *OIP5-AS1* levels. For instance, GAK localizes in the trans-Golgi network and functions as an Hsc70 cochaperone, playing essential roles in membrane trafficking during endocytosis and the uncoating of clathrin-coated vesicles [29, 30]. Interestingly, the influence of GAK on clathrin metabolism may be connected to its influence on mitosis, since clathrin is mobilized to the mitotic spindle and promotes microtubule stability in early mitosis [31]. Given that silencing clathrin impairs the assembly and segregation of chromosomes, causing arrest at metaphase, perhaps higher levels of GAK lead to the aberrant removal of clathrin and the same aberrant mitotic progression as that seen after silencing *OIP5-AS1*. The hypothesis that GAK overexpression might trigger aberrant mitoses and the additional possibility that this effect is due to the uncoating of clathrin from microtubules awaits experimental testing.

Other studies have shown that GAK is a transcriptional coactivator of the androgen receptor (AR), a ligand-dependent transcription factor highly expressed in prostate cancer [26]. In addition, GAK was shown to be a key player in the p53-MDM2 axis, helping to regulate p53 levels in cells responding to DNA damage stress [32]. Importantly, conditional depletion of GAK in mouse brain, liver or skin was lethal, even if the deletion was elicited in adult mice, indicating that GAK is essential both for embryonic development and for survival of adult animals [33]. By contrast, its transient knockdown induced DNA damage and misalignment of microtubule spindles in mitotic cells [25, 34], suggesting that long-term loss of GAK could be compensated during development by other proteins, while acute loss that did not allow adaptation was strongly cytotoxic.

The aberrant mitoses observed by silencing *OIP5-AS1* were due, at least to some extent, to the elevation of GAK in this transfection group, since concomitant silencing of GAK corrected this problem, restoring the mitotic phenotype close to that seen in the control population. At the same time, simply reducing GAK, as reported earlier [25], also caused aberrant mitoses including multipolar and monopolar spindles (Figure 5A). These sets of observations were somewhat unexpected, since they suggest that in both scenarios, when GAK is abnormally high and when GAK is abnormally low, cells exhibit impaired mitosis. Collectively, these findings suggest that GAK functions to orchestrate mitosis within a narrow range of abundance, and both excess or insufficient GAK impair mitotic progression.

In closing, the paradoxical function of GAK on cell division is further apparent when considering its impact on population growth. Short-term silencing of *OIP5-AS1* (for up to 5 days) elevated GAK and promoted cell proliferation, in agreement with higher production of cyclins A and D1 [23]. However, the burden of aberrant mitotic cells accumulating over time when GAK is elevated would be expected to prevent long-term expansion of the population. We propose that the complex impact of GAK on population growth depends on the specific context of the cell and whether the cell expresses proteins that enable senescence, death, proliferation or survival. The regulation of GAK levels by *OIP5-AS1* illustrates the dynamic impact of lncRNAs on post-transcriptional gene expression programs, including those that influence cancer and other diseases characterized by aberrant proliferation.

## MATERIALS AND METHODS

### Cell culture and transfection of siRNA and plasmids

Human cervical carcinoma HeLa cells and osteosarcoma U2OS cells were cultured in Dulbecco's modified Eagle's medium (DMEM) containing 10% (v/v) fetal bovine serum (FBS), antibiotics, and antimycotics. The siRNA duplexes listed in Supplementary Table 2 were transfected at 50 nM final concentration using Lipofectamine 2000 (Invitrogen). Plasmids (2 μg each) were transfected using Lipofectamine 2000.

### Total RNA and *MS2-OIP5-AS1*-bound RNA analysis

Total RNA was isolated from HeLa cells using Trizol (Invitrogen) and cDNA was synthesized by performing a conventional reverse transcription (RT) reaction using random hexamers and Maxima reverse transcriptase (Thermo Scientific). Following real-time, quantitative PCR (qPCR) amplification using specific primers (Supplementary Table 3) and SYBR green master mix (Kapa Biosystems) in an Applied Biosystems 7300 instrument, the relative levels of mRNAs of interest were measured by the 2^−ΔΔCt^ method.

The analysis of RNA bound to *MS2*-*OIP5-AS1* RNA was carried out using the MS2-RNA-tagging method described earlier [23]. Pulldown was performed using GSH-beads and RNA was labeled according to the manufacturer's instructions using the Illumina® TotalPrep^TM^ RNA amplification kit (Illumina, San Diego, CA). A total of 750 ng biotinylated aRNA was hybridized overnight to HumanHT-12 v4 BeadChip microarrays; after rinsing, arrays were incubated with streptavidin-conjugated Cy3, and scanned at 0.53 microns using an Illumina iScan scanner. Hybridization intensity data were extracted from the scanned images, and evaluated using Illumina GenomeStudio software, V2011.1. Raw and normalized microarray data are publicly accessible under GEO identifier GSE93551. Total RNA from synchronized U2OS cultures was analyzed by Arraystar (using AS-S-LNC-H microarrays).

### Western blot analysis

Total protein was lysed in RIPA buffer containing protease inhibitor and 1 mM dithiothreitol (DTT) on ice. Protein lysates were size-separated by SDS-PAGE and transferred onto nitrocellulose membrane (Invitrogen). For Western blot analysis, primary antibodies were used that recognized GAK (1:500) or HSP90 (1:20,000) (both from Santa Cruz Biotechnology) after blocking in 5% non-fat dry milk. After incubation with appropriate secondary antibodies, protein signals were detected using chemiluminescence (Millipore).

### Double thymidine block

For the double thymine block experiment, U2OS cells were cultured to 40% confluency in 10-mm dishes containing DMEM supplemented with 10% FBS. For the first thymidine block, cells were incubated in serum-free DMEM medium containing 2 mM thymidine (Sigma-Aldrich) and 14 h later cells were washed twice with PBS and incubated for 9 h in DMEM with 10% FBS; the second thymidine block was performed by repeating these same treatments on the same cells. After washing in PBS, cells were immediately harvested or further cultured in DMEM to reach the desired time points, whereupon cells were analyzed by fluorescence-activated cell sorting (FACS) using standard methods and RNA was extracted and quantified by microarray analysis.

### Antisense oligomer pulldown

For antisense oligomer pulldown analysis, streptavidin magnetic beads (Pierce) pre-washed in TENT buffer (10 mM Tris-HCl [pH 8.0], 1 mM EDTA [pH 8.0], 250 mM NaCl, 0.5% [v/v] Triton X-100, protease and RNase inhibitors) were incubated with sense or antisense biotinylated oligomers (Supplementary Table 4) for 30 min at room temperature. Cell lysates were prepared on ice in PEB buffer (20 mM Tris-HCl [pH 7.5], 100 mM KCl, 5 mM MgCl_2_, 0.5% Nonidet P-40) containing 1 mM DTT and protease and RNase inhibitors. The cell lysates were incubated with the beads coated with biotinylated sense or antisense oligomers for another 30 min at room temperature with gentle shaking. Using a magnetic column, the streptavidin beads were washed with TENT buffer, RNA was directly extracted from the beads using Trizol (Invitrogen) following the manufacturer's recommendations, and further analyzed by RT-qPCR to detect specific mRNAs bound to the biotinylated oligomers.

### DNA staining, immunostaining and microscopy

HeLa cells were cultured in 4-well chamber slides (Nalge Nunc). Seventy-two hours after transfection with the siRNAs indicated, cells were fixed in 4% paraformaldehyde and permeabilized in 1% Triton X-100. After washing with PBS three times, the cells were blocked in 4% bovine serum albumin (BSA) at room temperature for 1 h. The slides were incubated with primary antibodies recognizing α-tubulin (Sigma-Aldrich) or γ-tubulin (Santa Cruz) (1:400 dilution) followed by incubation with the appropriate fluorescent secondary antibodies. DNA was visualized by staining with Hoechst 33342 and the slide preparations were sealed and observed using an LSM 710 (ZEISS) microscope.

### Microarray and proteomic analyses

For the microarray analysis, RNAs bound to *OIP5-AS1* RNA were enriched by affinity pulldown using the MS2-tagging method described earlier [23]. Pulldown was performed using GSH beads and the bound RNA was analyzed using Illumina microarrays (GSE93551). Total RNA from synchronized U2OS cultures was analyzed by Arraystar (AS-S-LNC-H microarrays) (GSE93551).

For LC/MS analysis, total protein lysates were prepared from HeLa cells 72 h after transfection with Ctrl siRNA or *OIP5*-AS1 siRNA using RIPA buffer containing protease inhibitors on ice. Proteins were identified and analyzed by Poochon Scientific by employing a Q Exactive Mass Spectrometer and following the company protocol.

## SUPPLEMENTARY MATERIALS FIGURE AND TABLES




