# Supplementary material for: LncRNA OIP5-AS1/cyrano suppresses GAK expression to control mitosis
Source: Oncotarget. 2017 Apr 19;8(30):49409–20. doi: 10.18632/oncotarget.17219 (PMC5564778; doi:10.18632/oncotarget.17219)
Supplement: Supplementary file 1 [file oncotarget-08-49409-s001.pdf]

LncRNA *OIP5-AS1/cyrano* suppresses GAK expression to control mitosis

SUPPLEMENTARY MATERIALS

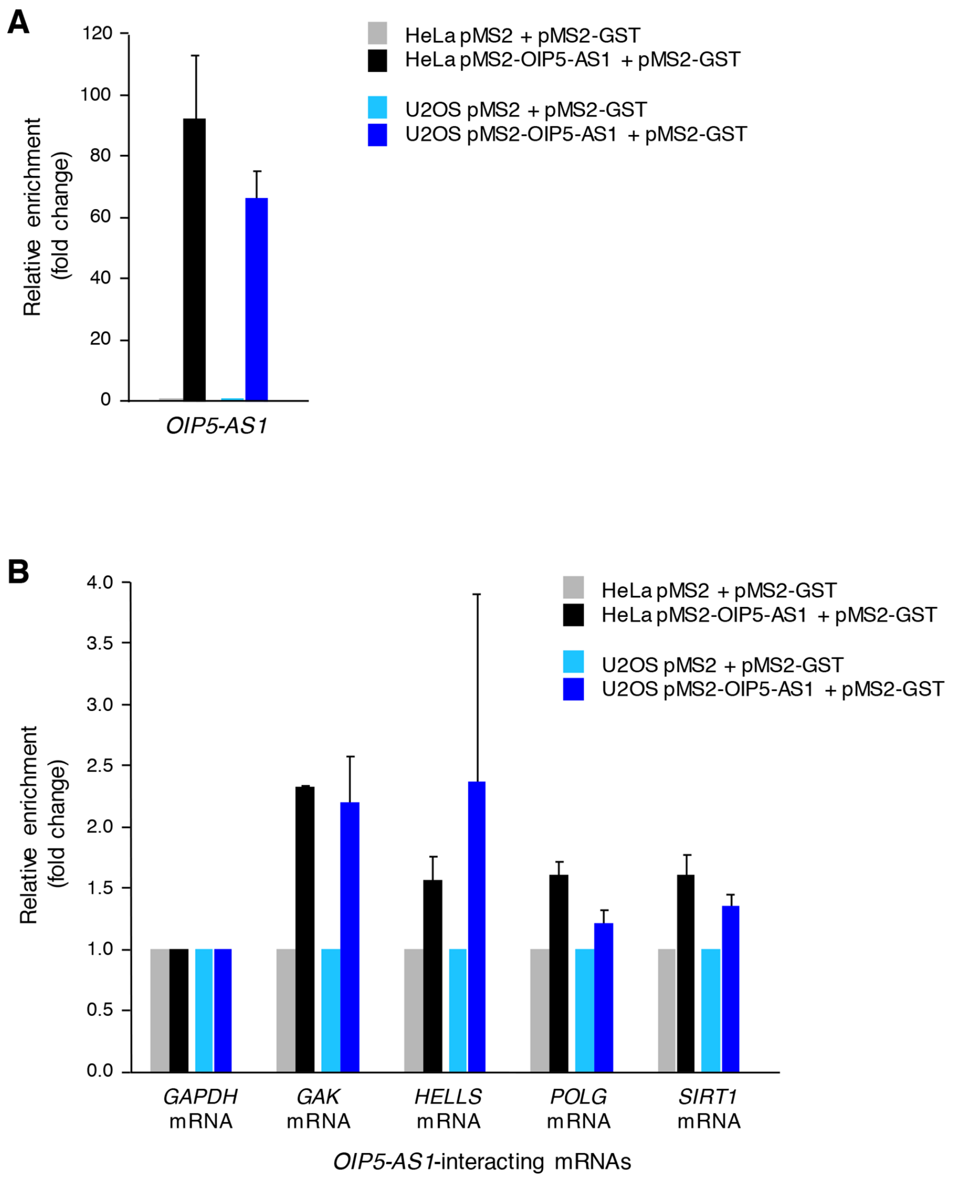

Supplementary Figure 1: Assessment of the interaction of *OIP5-AS1* with target mRNAs in HeLa and U2OS cells using the MS2 RNA-tagging method. (A) HeLa cells and U2OS cells were transfected with two expression vectors, one that transcribed the chimeric RNA *MS2-OIP5-AS1* (or a control vector that only expressed the *MS2* RNA hairpins) and one that expressed a MS2-GST fusion protein. GSH beads were then used to pull down the MS2 complex from cell lysates, thereby capturing *MS2-OIP5-AS1* and molecules bound to it. Associated mRNAs were then identified by RT-qPCR analysis, including a negative control (*GAPDH* mRNA), a positive control (*GAK* mRNA), and other mRNAs that were found enriched by microarray analysis. Data are the means + S.E.M. from three independent experiments.

Supplementary Table 1:

See Supplementary File 1

Supplementary Table 2: siRNA duplexes used in this study

siRNA (Provider)	Sequence
Ctrl siRNA (Qiagen)	AATTCTCCGAACGTGTCACGT
OIP5-AS1 (IDT)	Sense1 rGrGrCrUrGrArGrUrUrUrCrArUrUrUrGrArArArCrArGrGTG<br>Antisense1 rCrArCrCrUrGrUrUrUrCrArArArUrGrArArArCrUrCrArGrCrCrUrU<br>Sense2 rCrArUrGrCrArGrUrGrCrCrArUrCrUrGrArCrUrUrUrArUGG<br>Antisense2 rCrCrArUrArArArGrUrCrArGrArUrGrGrCrArCrUrGrCrArUrGrArG<br>Sense3 rCrArCrCrArArArCrArGrGrCrUrUrUrGrUrGrUrUrCrCrUTA<br>Antisense3 rUrArArGrGrArArCrArCrArArArGrCrCrUrGrUrUrUrGrGrUrGrGrU
GAK (IDT)	Sense1 rGrArGrArArCrUrUrGrUrUrGrCrUrUrArGrUrArArCrCrAAG<br>Antisense1 rCrUrUrGrGrUrUrArCrUrArArGrCrArArCrArArGrUrUrCrUrCrArA<br>Sense2 rGrUrUrUrUrG rUrUrCrUrGrCrArGrCrGrUrCrUrArUrArGGA<br>Antisense2 rUrCrCrUrArUrArGrArCrGrCrUrGrCrArGrArArCrArArArArCrUrG

Supplementary Table 3: Primers for qPCR amplification

Oligomers	Sequence
OIP5-AS1-F	TGCGAAGATGGCGGAGTAAG
OIP5-AS1-R	TAGTTCCTCTCCTCTGGCCG
GAK-F	CACCAACCTCAAGGACACCT
GAK-R	TGACTCCACACCTTCTGCTG
GAK-2-F	CTGCAGGTGAACCCGGAGGAGC
GAK-2-R	CCAAGCTTCTAATACGACTCACTATAGGGAGAACCCCGTGTGGAACTGAATCTGGA
HELLS-F	AGAGGTCACAAGGTGCTGCT
HELLS-R	AAGACATGGACCCATCAAGC
POLG-F	CAACCCCTAGCTCTGACTGC
POLG-R	GAGGCAGCTTGAAAAACCAG
SIRT1-F	TCAGTGGCTGGAACAGTGAG
SIRT1-R	AGCGCCATGGAAAATGTAAC
GAPDH-F	TGCACCACCAACTGCTTAGC
GAPDH-R	GGCATGGACTGTGGTCATGAG
18S-F	CGAACGTCTGCCCTATCAACTT
18S-R	ACCCGTGGTCACCATGGTA

Forward (F) and reverse (R) primers used in this study are named by the transcript they amplify.

Supplementary Table 4: DNA oligomers

DNA Oligomers	Sequence
GAK-biotin-AS1	GCTGGTGCACCACCTCGGCGAT/3'biotin
GAK-biotin-AS2	ACCCCGTGTGGAACTGAATCTGGA/3'biotin
GAK-biotin-AS3	GCAGTCTCGGTCCAGGCAGCCCAT/3'biotin
GAK-biotin-AS4	GCGGTGCAAACACCAGGGCCCATG/3'biotin
GAK-biotin-S1	ATCGCCGAGGTGGTGCACCAGC/3'biotin
GAK-biotin-S2	TCCAGATTCAGTTCCACACGGGGT/3'biotin
GAK-biotin-S3	ATGGGCTGCCTGGACCGAGACTGC/3'biotin
GAK-biotin-S4	CATGGGCCCTGGTGTTTGCACCGC/3'biotin
OIP5-AS1-biotin-AS1	CCGATATTTTTTTTCTTCTC/3'biotin
OIP5-AS1-biotin-AS2	GTTGAATTTGAGAGGCAGAAC/3'biotin
OIP5-AS1-biotin-AS3	TAGTATCTTTCACGTCAAAT/3'biotin
OIP5-AS1-biotin-AS4	ATTGTAATTATTTTTCTTAT/3'biotin
OIP5-AS1-biotin-S1	GAGAAGAAAAAAAATATCGG/3'biotin
OIP5-AS1-biotin-S2	GTTCTGCCTCTCAAATTCAAC/3'biotin
OIP5-AS1-biotin-S3	ATTTGACGTGAAAGATACTA/3'biotin
OIP5-AS1-biotin-S4	ATAAGAAAAATAATTACAAT/3'biotin

Sense (S) and antisense (AS) biotinylated oligomers used to pull down *GAK* mRNA and *OIP5-AS1*.
